# The MADS-box gene *EjAGL15* positively regulates lignin deposition in the flesh of loquat fruit during its storage

**DOI:** 10.3389/fpls.2023.1166262

**Published:** 2023-05-10

**Authors:** Hang Ge, Hongxia Xu, Xiaoying Li, Junwei Chen

**Affiliations:** Institute of Horticulture, Zhejiang Academy of Agricultural Sciences, Hangzhou, Zhejiang, China

**Keywords:** loquat, lignin, MADS-box, postharvest storage, senescence

## Abstract

**Introduction:**

Lignification of fruit flesh is a common physiological disorder that occurs during post-harvest storage, resulting in the deterioration of fruit quality. Lignin deposition in loquat fruit flesh occurs due to chilling injury or senescence, at temperatures around 0°C or 20°C, respectively. Despite extensive research on the molecular mechanisms underlying chilling-induced lignification, the key genes responsible for the lignification process during senescence in loquat fruit remain unknown. MADS-box genes, an evolutionarily conserved transcription factor family, have been suggested to play a role in regulating senescence. However, it is still unclear whether MADS-box genes can regulate the lignin deposition that arises from fruit senescence.

**Methods:**

Both senescence- and chilling-induced flesh lignification were simulated by applying temperature treatments on loquat fruits. The flesh lignin content during the storage was measured. Transcriptomic, quantitative reverse transcription PCR and correlation analysis were employed to identify key MADS-box genes that may be involved in flesh lignification. The Dual-luciferase assay was utilized to identify the potential interactions between MADS-box members and genes in phenylpropanoid pathway.

**Results and Discussion:**

The lignin content of the flesh samples treated at 20°C or 0°C increased during storage, but at different rates. Results from transcriptome analysis, quantitative reverse transcription PCR, and correlation analysis led us to identify a senescence-specific MADS-box gene, EjAGL15, which correlated positively with the variation in lignin content of loquat fruit. Luciferase assay results confirmed that EjAGL15 activated multiple lignin biosynthesis-related genes. Our findings suggest that EjAGL15 functions as a positive regulator of senescence-induced flesh lignification in loquat fruit.

## 1 Introduction

Loquat fruit (*Eriobotrya japonica* Lindl.) has a delicious taste and is rich in carotenoids, two features which make it popular among consumers in Asia and Europe. However, the taste of loquat fruit rapidly deteriorates once harvested if it is stored at room temperature, which reduces the feasibility of both its long-term storage and long-distance transportation. The flesh of loquat fruit becomes less juicy and harder to chew when such postharvest deterioration takes place (Cai et al., 2006a). Recent research has clarified that the deposition of lignin in loquat flesh is the leading cause for its diminished taste (Huang et al., 2019). Accordingly, developing effective and inexpensive methods to alleviate the fruity flesh lignification is imperative for securing and promoting the loquat industry.

In the last decade, cold chain has been gradually applied to the transportation and storage of fruits and vegetables, significantly delaying their postharvest spoilage via the precise management of air temperature and suppression of senescence (Mercier et al., 2017; Yang et al., 2020). Yet fruits can also often exhibit physiological disorders, such as internal browning and loss of aromatic flavors, when they undergo inappropriate cold storage (Ding et al., 2002; Zheng et al., 2022). Unfortunately, loquat fruit not only exhibits all the above symptoms but also incurs lignin deposition in its flesh when the temperature drops below its threshold for chilling injury (Cai et al., 2006c), which greatly limits the application of cold chain technology. Hence, suitable conditions that would not cause chilling injury are needed to maintain the commodity properties (and thus market value) of loquat fruits. Nevertheless, the lignification of loquat flesh could also be advanced by senescence under room temperature conditions (Yang et al., 2010), which complicates the optimization of a suitable storage temperature range. Consequently, both chilling and senescence effects should be taking into consideration when trying to balance the benefits versus losses of using cold chain storage.

To precisely define the temperature that best minimizes or stalls the lignification of loquat’s flesh during its storage, the molecular mechanism by which flesh is lignified should be first elucidated. Key genes that regulate the deposition of lignin require robust identification for guiding the invention of convenient and inexpensive storage methods. Current studies on loquat lignification have revealed that several genes account for chilling-induced lignin deposition, together with the conditions that initial their transcription, which provides a promising basis to estimate the optimal temperature interval. For example, *EjMYB1*, *EjNAC3*, and *EjHAT1* participate in lignin biosynthesis by interacting with genes in the phenylpropanoid pathway, and their transcripts at 0°C are more abundant than that at 5°C, consistent with a chilling-induced expression pattern (Xu et al., 2014; Ge et al., 2017; Xu et al., 2019). Nonetheless, comparatively little attention has been paid to senescence- induced lignification. Although the abnormal deposition of lignin during fruit senescence has been attributed to the activation of enzymes in phenylpropanoid pathway like Phenylalanine ammonia-lyase (PAL) and Cinnamyl alcohol dehydrogenase (CAD) which is identical to that during chilling injury (Cai et al., 2006b; Chong et al., 2006; Li et al., 2017), the mechanism underlying the temperature-dependent regulation of that pathway remains poorly clarified with an insufficient characterization of the key regulators involved. It was recently shown that multiple regulators are recruited when chilling-induced lignification occurs (Shi et al., 2022), but none of them have been proven to play roles in the lignification process at room temperature. In fact, the transcripts of *EjERF39* and *EjMYB8*, two vital regulators of chilling-induced lignification, remain at low abundance during loquat fruit’s storage at room temperature (Zhang et al., 2020). These findings imply the existence of a unique regulation pathway underpinning senescence-induced lignification that differs from chilling-induced lignification.

Lignin deposition is a process during plant development that is mainly controlled by NAC and MYB transcription factors, known as master switches of the secondary cell wall (Wang et al., 2011; Zhong and Ye, 2012; Nakano et al., 2015). However, regulation of stress-induced lignification in different plant tissues is believed to entail novel players other than NAC and MYB (Cesarino, 2019). MADS-box is a conserved gene family found in plants as well as animals. Many studies have presented evidence for linkages between MADS-box members and senescence in different plant species; for example, *Arabidopsis thaliana* (Fang and Fernandez, 2002), *Solanum lycopersicum* (Xie et al., 2014), *Medicago truncatula* (De Michele et al., 2009), and *Brassica rapa* (Yi et al., 2021). The overexpression of MADS-box genes usually results in a delayed senescence process in different organs, which demonstrates the role of negative regulators of senescence. During senescence, lignin deposition is activated in certain kinds of fruit and vegetables besides loquat, such as in wax apple (Hao et al., 2016) and bamboo (Li et al., 2019). But it is unclear whether tissue lignification process during senescence is controlled by MADS-box genes. Interestingly, recent studies found that MADS-box genes are capable of controlling lignin deposition in specific plant tissues such as the stem (Cosio et al., 2017) and fruit flesh (Ge et al., 2021), which expands the scope of MADS-box genes’ molecular functioning. By synthesizing the available information, a hypothesis may be deduced: that MADS-box genes are somehow involved in senescence-triggered lignification in loquat flesh.

In this study, both senescence- and chilling-induced lignification of flesh during the storage of loquat fruits were simulated by temperature treatments. The lignin content of flesh samples under two treatments, 20°C and 0°C, was measured. Transcriptome and correlation analysis were carried out to distinguish the MADS-box genes associated with senescence-induced lignification; a MADS-box gene, named *EjAGL15*, was found to be positively related to the variation in lignin content of loquat flesh. Meanwhile, the biological function of the identified MADS-box gene for regulating lignin deposition was investigated via the dual luciferase assay.

## 2 Materials and methods

### 2.1 Plant materials and treatment

Loquat fruits (*E. japonica* cultivar ‘Luoyangqing’) were harvested in Luqiao, Zhejiang Province, China, then packaged in plastic foam boxes, and taken to the laboratory by vehicle. Upon arrival the fruits were immediately sorted and selected according to the color of their pericarp, ensuring uniformity of maturity, and transferred to atmosphere chambers. Two chambers were set respectively to 20°C and 0°C, as two treatment groups, thus emulating shelf storage and cold storage conditions, respectively. Fruits were sampled at the beginning of storage, and then again after 2, 6, and 10 days. Each time-point had three biological replicates, with each replicate consisting of four fruits. These fruit flesh samples without the pericarp were immediately frozen in liquid nitrogen for status fixation, then stored at –80°C.

### 2.2 Lignin content analysis

Total lignin content was measured based on the methodology described by Lowry et al. (1994) with minor modifications applied. The 1-g lyophilized sample was ground into powder and mixed with 100 mL of acidic wash buffer (0.5 mol/L sulfuric acid, 0.05 mol/L hexadecyl trimethyl ammonium Bromide), 2 mL of decahydronaphthalene, and 0.5 g of sodium sulfite in a beaker, then boiled for 60 min. After discarding the acidic wash buffer, the residual material was washed with distilled water until the flow-through became neutral; this was followed by another washing step using about 20 mL of acetone. The washed residual material was transferred into an oven and dried at 105°C for 2 h. This dried residue, which mainly consisted of acid detergent fiber (ADF), was put in a desiccator for 30 min, and cooled to room temperature. The ADF was hydrolyzed in 10 mL of 72% H_2_SO_4_ for 3 h then incubated overnight after adding 45 mL of distilled water. The hydrolyzed ADF was weighed (W_1_) following filtration and a washing step. The residual material was transferred to a muffle furnace and burned for 2.5 h at 550°C, and then weighed (W_2_). The lignin content was equal to the weight loss (W_1_–W_2_) of the residual material.

### 2.3 Detection of guaiacyl and syringyl lignin units

The 20-mg well-ground sample was mixed with 1 mL of a freshly prepared dioxane solution containing 2.5% boron trifluoride and 10% ethyl mercaptan. The mixture was then incubated at 100°C for 4 h, and the reaction ended by rapid cooling at –20°C for 5 min. Tetracosane was added, as an internal standard, to yield final concentration of 0.1 mg/mL. Meanwhile, each sample was thoroughly mixed with dichloromethane (1 mL) and water (2 mL) for separation. The organic phase was carefully transferred to a new tube into which sodium sulfate was added to remove the water. After complete volatilization, the solute was redissolved in 0.4 mL of dichloromethane. Before its gas chromatography analysis, pyrimidine (50 μL) and bovine serum albumin (100 μL) were added to the sample solution, and it incubated at 25°C for 4 h.

The degraded lignin units were determined by GC/MS (Trace1310 ISQ, Thermo) with a TG-5MS column (Thermo, 30 m × 0.25 mm × 0.25 μm). The GC conditions were set as follows: initial column temperature of 60°C; ramped at 35°C/min, 0.5°C/min, and 50°C/min to 220°C, 230°C, and 280°C respectively, then held for 7 min; injection temperature of 250°C; flow rate of carrier gas: 1.2 mL/min; sample loading volume of 2 μL; injection model: split, using a split ratio of 20:1. The MS conditions were as follows: an EI voltage of 70 eV; ion source temperature of 200°C; interface temperature of 250°C; solvent delay, 5 min; and, monitoring range: 40–650 amu.

### 2.4 Total RNA extraction and quantitative reverse transcription PCR

Total RNA in the flesh of each replicate sample was extracted using CTAB methods (Shan et al., 2008). Genomic DNA was removed by implementing the TURBO DNA-free kit (Ambion). The quality of extracted RNA was determined by gel electrophoresis and spectrophotometry (Implen), after which the corresponding first-strand cDNA was synthesized using the PrimeScript™ RT reagent kit (Takara). The gene transcripts were quantified on a CFX384 Real-Time System (Biorad), with SsoFast EvaGreen Supermix (Biorad). The primers for quantitative reverse transcription PCR were designed based on the 3’ region of coding sequence, using Primer3 software (v4.0.0; http://bioinfo.ut.ee/primer3/), and are listed in **Supplementary Table S1**. The PCR program was set to 30 s at 95°C, followed by 45 cycles at 95°C for 5 s and 60°C for 5 s, and completed with a melting curve analysis program. The *EjACT* (Fu et al., 2012) gene served as an internal control and relative gene expression levels were calculated using the 2^–ΔCT^ method.

### 2.5 Phylogenetic analysis

The amino acid sequences of Arabidopsis and loquat MADS-box genes were respectively downloaded from TAIR (www.arabidopsis.org) and NCBI (www.ncbi.nlm.nih.gov). The phylogenetic tree was constructed using MEGA 11 (Tamura et al., 2021), by applying the Neighbor-Join and BioNJ algorithms to a matrix of pairwise distances estimated using the JTT (Jones-Taylor-Thornton) model, and then selecting the topology having the superior log-likelihood value. A discrete Gamma distribution was used to model the differences in evolutionary rate among sites. The branches and gene names were then labeled with different colors, using the ‘ggtree’ (Yu et al., 2017) and ‘treeio’ (Wang et al., 2020) R packages.

### 2.6 Dual luciferase assay

The dual luciferase assay was performed as previously described (Xu et al., 2014). The coding sequence of *EjAGL15* was amplified using the primer listed in **Supplementary Table S2** and inserted into the pGreen II 0029 62-SK vector (SK). The promoters of lignin deposition-related genes from both loquat and Arabidopsis were delivered to the pGreen II 0800-LUC vector (LUC). All the recombinant SK and LUC vectors were individually transfected into *Agrobacterium tumefaciens* GV3101. The glycerol stocks with transfected *Agrobacterium* were grown in a lysogeny broth (LB) plates with kanamycin (50 μg/mL) and gentamycin (25 μg/mL) for 2 days, and then restreaked onto new LB plates for 1 day. *Agrobacterium* cells were suspended in an infiltration buffer (10 mM MES, 10 mM MgCl_2_, 150 mM acetosyringone, pH 5.6) to an optimal density (OD_600_ = 0.75), then 1 mL of *Agrobacterium* cultures containing *EjAGL15* were mixed with 100 μL of *Agrobacterium* containing the promoters. The mixtures were then injected into tobacco (*Nicotiana tabacum*) leaves, via needleless syringes; 3 days after that infiltration, both LUC and REN fluorescence intensities were measured using the dual luciferase assay reagents (Promega). Five replicates were conducted for *EjAGL15* and each promoter combination.

### 2.7 Statistical analyses

Pearson’s correlation coefficient and *p-*values between the transcript abundance of MADS-box genes and lignin content flesh were first calculated and then arranged in the form of a matrix, using R software (v4.2.2). This matrix was then visualized using the R package ‘corrplot’. Statistical differences were tested by two-tailed, unpaired Student’s *t*-test and ANOVA followed by Fisher’s LSD test using Origin 2023. For all these analyses, a significance level *p* < 0.05 was used.

## 3 Results

### 3.1 Distinct pattern of lignin accumulation under contrasting temperatures

**Figure 1:**
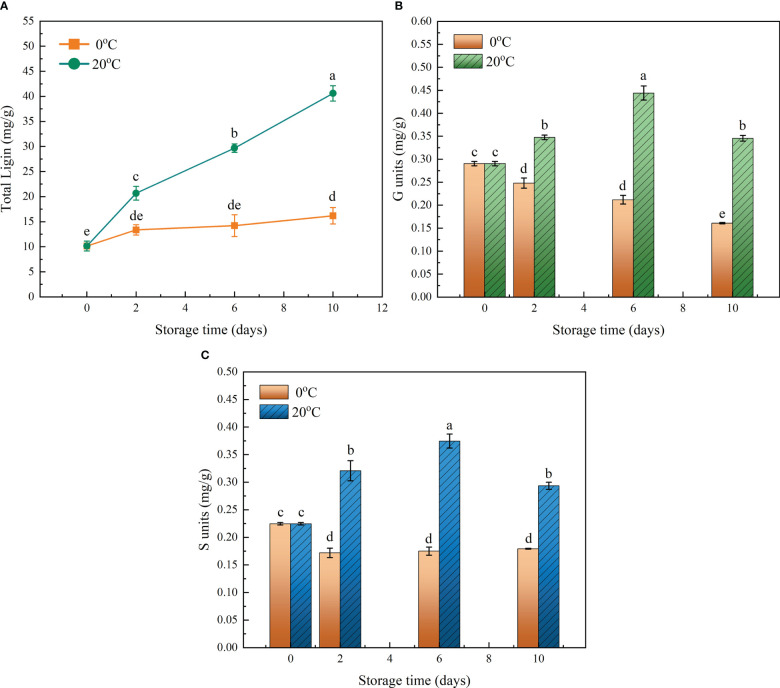
Effects of two contrasting storage temperatures on the lignin content of flesh in loquat fruit during its storage. **(A)** Change of total lignin content during the storage period. **(B)** Content of the guaiacyl (G) lignin unit. **(C)** Content of the syringyl (S) lignin unit. Error bars are the SE of the mean, based on n = 3 biological replicates. Different letters indicate significantly different means of groups in the comparison (*p* < 0.05).

The amounts of total lignin and the three units consisting of differing monolignols in the flesh of loquat fruits were monitored during their storage under the 20°C and 0°C treatments. The fruits stored at 20°C continuously accumulated lignin in flesh during the whole storage period, increasing their content from 10.15 to 40.6 mg/g, a phenomenon typical of postharvest flesh lignification. On the contrary, lignin accumulation was effectively alleviated by storage at 0°C, though the lignin content did slowly increase from 10.15 to 16.18 mg/g due to chilling injury (**Figure 1A**). As a kind of dicot plant, loquat’s fruit flesh contains the guaiacyl (G) and syringyl (S) lignin units, but the *p*-hydroxyphenyl (H) lignin unit was beyond the detection limit (**Figures 1B, C**). The content of both G and S units changed in a temperature-dependent manner, which again confirmed the stalled lignification by cold storage. The greatest difference in lignin units arose on day 6 of storage, when the G and S units were 52.9% and 66.7% higher than at the onset of storage, respectively, indicating the continuous activation of the lignin biosynthesis pathway.

### 3.2 Quantified transcripts of MADS-box family genes

**Figure 2:**
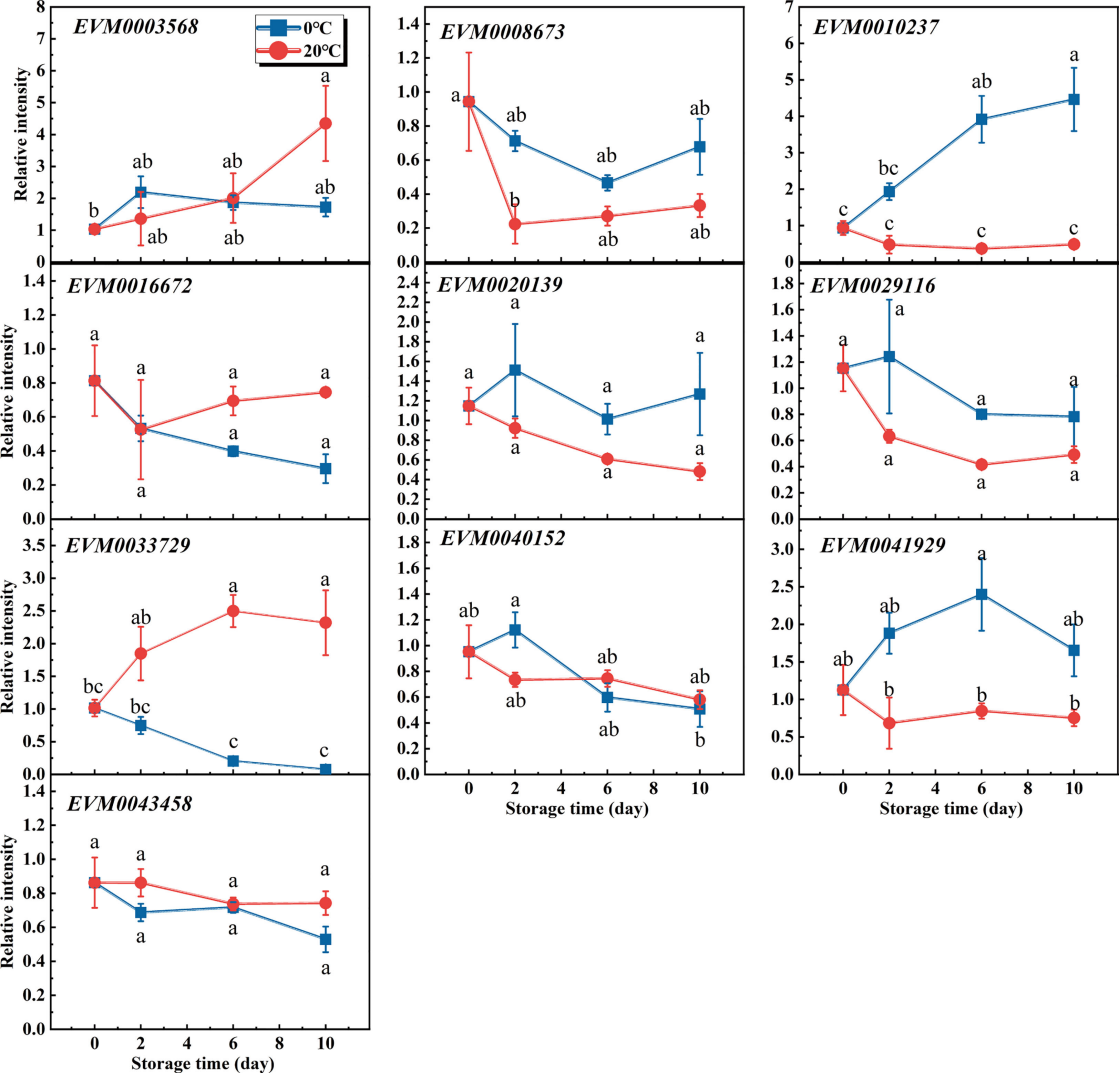
Characterization of the differentially expressed MADS-box genes by quantitative reverse transcription PCR and the transcriptome analysis. Transcripts of 10 MADS-box genes were monitored during the storage of loquat fruit under 20°C versus 0°C. Error bars are the SE of the mean, based on n = 3 biological replicates. Different letters indicate significantly different means of groups in the comparison (*p* < 0.05).

Because the lignin content of flesh differed significantly between the 20°C and 0°C conditions at 6 days of storage, samples from two time-points (the 1st and 6th day under storage) of each condition were collected for their transcriptome analysis. Although about 89 MADS-box members were annotated in the loquat genome database (Liu et al., 2022), we found only 28 MADS-box genes active above the detection limit of high-throughput sequencing. Next, we tried to trace the transcripts of those MADS-box genes detected in the transcriptome data at all time-points during the storage experiment, as this could provide more information. Due to the low abundance of transcripts, 10 pairs of primers met the requirements for successful measurements. The changed transcript levels of loquat’s MADS-box genes during the storage period are shown in **Figure 2**. Despite all these genes being expressed differently between the two time-points under the same storage condition, or between the two storage temperatures within the same time-point based on transcriptome data, some of them were statistically insignificant. Actually, the transcript abundances of *EVM0016672*, *EVM0029116*, *EVM0043458*, and *EVM0020139* were not significantly different at any time-points within or between storage conditions, whereas *EVM003568*, *EVM0040152*, and *EVM0068673* were expressed differently only at time-points of a certain storage condition. Additionally, the transcripts of *EVM0041929* and *EVM0010237* were induced in loquat fruits during their storage under 0°C but maintained at a stable level under 20°C. Meanwhile, their difference in transcripts between temperatures was significant when fruits were stored for 6 days or longer. In contrast to *EVM0041929* and *EVM0010237*, the transcript abundance of *EVM0033729* continually increased at 20°C, significantly exceeding that at 0°C after 6 days of storage (**Figure 2**). The differential responses to temperature of loquat MADS-box genes may point to their distinctive functions in the lignification process under different storage conditions. However, further analysis of differentially expressed MADS-box genes (DEGs) using transcriptome data revealed four genes which exceeded the threshold of both the fold change and *p*-value between the comparison groups (**Supplementary Figures 1A–C**), of which *EVM0033729* was the only gene differentially expressed in all comparison groups (**Supplementary Figure 1D**). Thus, *EVM0033729* exhibited a unique temperature-dependent expression pattern in loquat flesh during fruit storage, which suggested it was highly involved in the lignification process.

### 3.3 Phylogenetic analysis of MADS-box genes

The sequences of the 10 quantified MADS-box genes were analyzed according to the Conserved Domain Database (https://www.ncbi.nlm.nih.gov/Structure/cdd/cdd.shtml), and all of them scored hits with specific motifs. All sequences harbored the conserved MADS-box domain at their N terminal (**Supplementary Figure 2**), which is a homolog to the SRF domain in mammals. But three of the sequences lacked the K domain, as symbolized in the MIKC subgroup of the MADS-box family. The gene structure of each of these MADS-box genes was visualized next, according to the genome data. Genes with or without the K domain differ in the number and length of introns (**Supplementary Figure 3**). Generally, genes without the K domain have fewer or shorter introns than genes with the K domain. Even *EVM0008673* had no introns, implying the conservation of its gene functions during the evolution of loquat plants.

**Figure 3:**
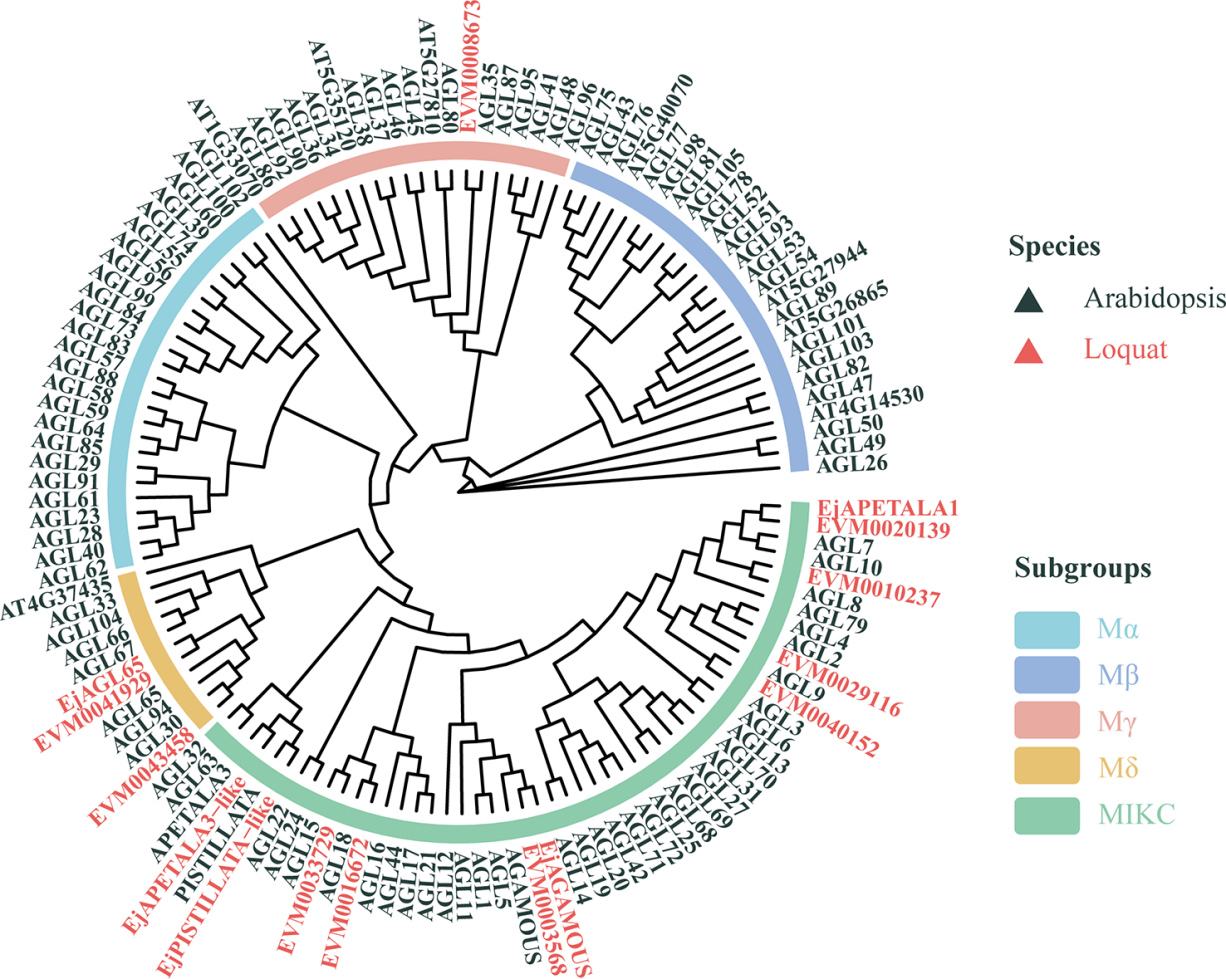
Phylogenetic analysis of loquat and Arabidopsis MADS-box genes. The tree was constructed using the Jones-Taylor-Thornton model. Genes belonging to loquat and Arabidopsis are in red and black, respectively. Arcs with different colors represent the five subgroups of Arabidopsis MADS-box genes.

To better predict their biological function, we built a phylogenetic tree using the sequences of the 10 MADS-box genes, together with four previously reported loquat MADS-box genes and Arabidopsis MADS-box analogs, whose functions are well elucidated. Multiple models for describing the substitution pattern were first evaluated and the JTT with a discrete Gamma distribution (JTT+G+F) mixed model yielded the lowest Bayesian Information Criterion (BIC) score (**Supplementary Table S3**). As **Figure 3** shows, the Arabidopsis MADS-box genes were divided into five clades (Mα, Mβ, Mγ, Mδ, MIKC), which is consistent with the findings of an earlier report (Parenicova et al., 2003). Different colors were used to distinguish species and subgroups to enhance their visualization. Evidently, the loquat MADS-box genes are dispersed in a different subgroup of the tree, but the number of loquat genes in each clade varies considerably. Most loquat MADS-box genes are localized at the MIKC group, while the Mα, Mβ, Mγ, and Mδ group included 0, 0, 1, and 2 genes, respectively. This constructed tree provided general structural and evolutionary information about loquat’s MADS-box genes extracted from its genome, but clues concerning lignin-related MADS-box gene were limited. The MADS-box members are mainly considered as key regulators of flowering; hence, the relation between MADS-box genes and lignin content remains poorly elucidated. In fact, Arabidopsis AGL15 is the only member confirmed as involved in lignin deposition (Cosio et al., 2017). Thus, EVM0033729 may have a similar function to AGL15 due to their short evolutionary distance and was renamed *EjAGL15* for the follow-up investigation.

### 3.4 Relationship between *EjAGL15* transcripts and lignin content during storage

**Figure 4:**
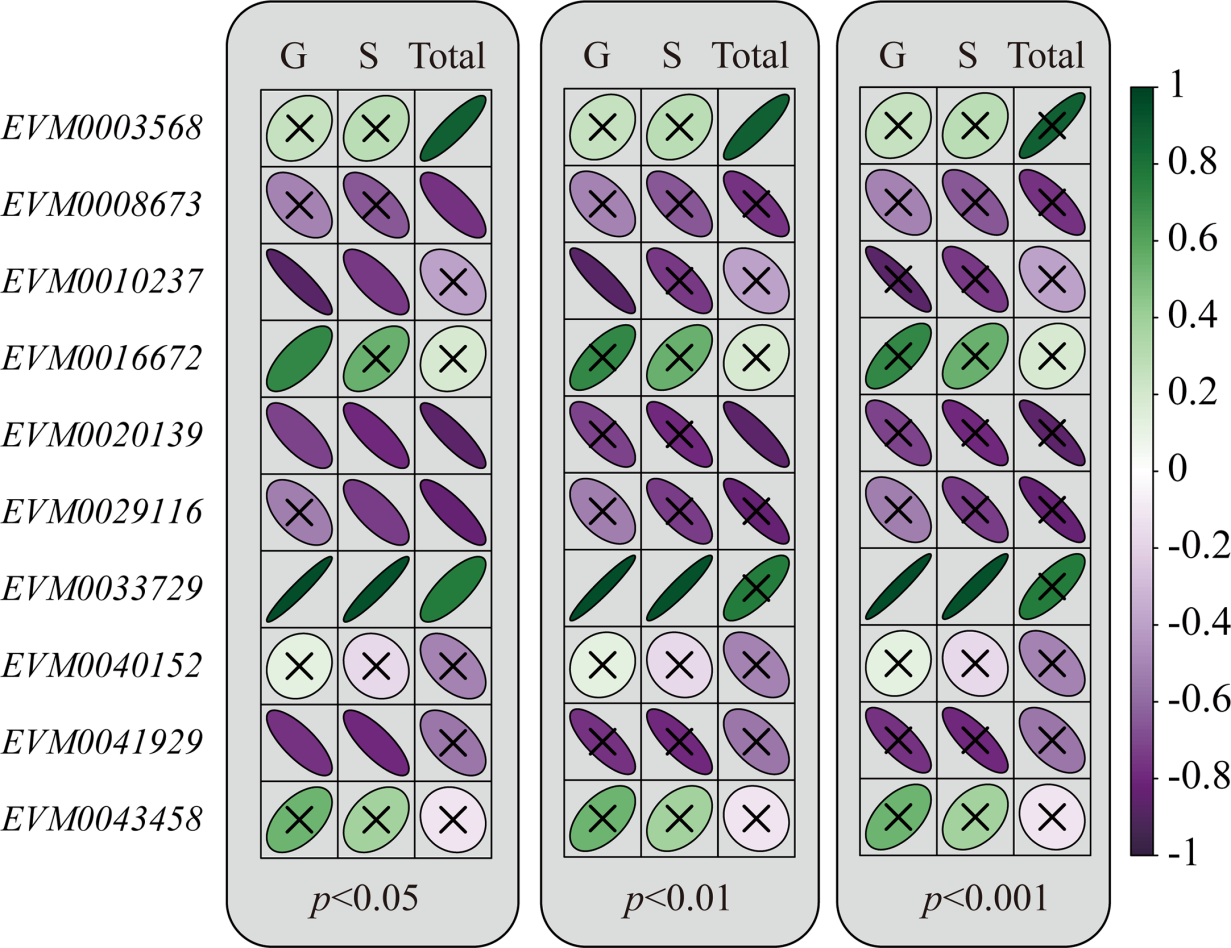
Relationship between the change in transcripts abundance and lignin content during the storage of loquat fruit. The Pearson correlation coefficients are visualized by the color gradient (far right). Green color indicates a positive value while purple refers to a negative value for Pearson’s *r*. The significance of each correlation was visualized by the focal distance of ellipse. A flatter ellipse represents a lower *p*-value for the correlation. Insignificant correlations under a certain significant level are labeled with the cross symbol.

Correlations were tested given that the transcript levels of MADS-box genes and the lignin content of flesh both varied in a temperature-dependent pattern. Genes except *EVM0043458* and *EVM0040152* had a positive or negative correlation with either the content of monolignols or total lignin (**Figure 4** all *p*-values < 0.05). To obtain more convincing results, the correlations were re-evaluated using a lower threshold *p*-value. Correspondingly, we uncovered five highly significant correlations (all *p*-values < 0.01). Specifically, *EVM0010237* and *EVM0020139* were negatively correlated with the G unit and total lignin content, respectively, while *EVM0033729* and *EVM0003568* had positive correlations with the either G unit, S unit, or total lignin content. Moreover, when applying a stricter threshold of *p* < 0.001, the correlation between *EVM0033729* and the G or S unit were still significant. Besides, among these MADS-box genes, the coefficient values for the correlations of *EVM0033729* were the highest (*r* = 0.81 and *r* = 0.79 with the G and S unit, respectively). These empirical clues strongly implied that a close relationship between *EjAGL15* and lignin deposition exists in the flesh of loquat fruit.

### 3.5 Interactions between EjAGL15 and lignin biosynthesis-related genes

**Figure 5:**
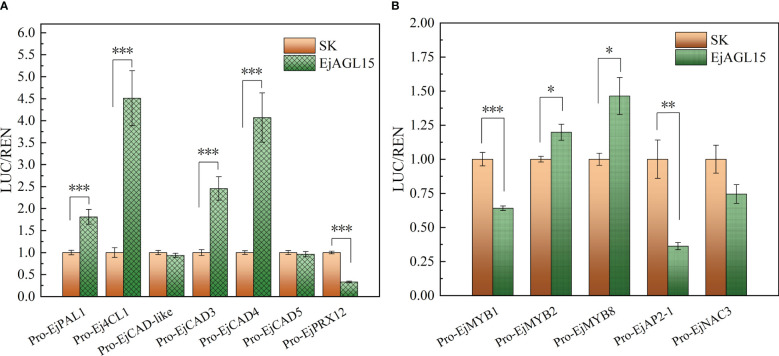
Biological effect of EjAGL15 on the promoter region of genes in the phenylpropanoid pathway **(A)** and lignification-related transcription factors **(B)**. Error bars are the SE of means based on five biological replicates. The number of asterisks corresponds to a more stringent significant level (Student’s *t*-test; "*", "**" and "***" mean p<0.05, 0.01 and 0.001 respectively).

Altogether, the above results pinpointed a MADS-box gene, *EjAGL15*, as being likely involved in regulating the lignification process in fleshy tissues of loquat. Next, the dual luciferase assay was performed to reveal the potential protein-DNA interactions *in vivo*. As **Figure 5A** shows, EjAGL15 had a strong activation effect on the promoters of four genes: *EjPAL1*, *Ej4CL1*, *EjCAD3*, and *EjCAD4*. In addition to the induction of genes participating in the lignin biosynthesis pathway, *EjAGL15* also significantly activated certain transcriptional regulators, like *EjMYB8*, which is a key activator of flesh lignin deposition, suggesting its role as a positive regulator. However, another activator, *EjMYB1*, was inhibited by transient overexpression of *EjAGL15*; this runs counter to an increased lignin content of flesh. Meanwhile, activation of the *EjMYB2*’s promoter could have a negative influence on the rate of lignin deposition as well. Further, the expression of *EjPRX12*, which is known to be involved in the polymerization of monolignols (Zhang et al., 2022), is inhibited by EjAGL15. This inconsistent influence on positive or negative regulators of the flesh lignification process means that *EjAGL15* probably fulfills complex roles in the manipulation of lignin deposition in loquat fruit during the senescence process.

## 4 Discussion

### 4.1 *EjAGL15* positively modulates lignin deposition in loquat flesh

Fruits generally contain less lignin when compared to other parts of plants. However, the fruit of loquat (*E. japonica*) accumulates lignin in its flesh during postharvest storage, being a typical example of fruit lignification. Extensive studies have focused on revealing the molecular mechanism behind flesh lignification. A series of transcription factors have been characterized as regulators of flesh lignification in loquat, notably the MYB (Xu et al., 2014; Zhang et al., 2020) and NAC (Ge et al., 2017) family members. Recently, novel regulators other than NAC and MYB have been uncovered, providing new perspectives and insight for understanding the complicated regulatory mechanism of fruity flesh lignification (Xu et al., 2019; Ge et al., 2021; Zhang et al., 2022). Here, we found that the MADS-box family gene *EjAGL15* is involved in lignin deposition in fruit flesh. Unlike other regulators of flesh lignification, *EjAGL15* is an activator of multiple genes in the phenylpropanoid pathway, including *EjPAL1*, *Ej4CL1*, *EjCAD3*, and *EjCAD4*. Both *EjPAL1* and *Ej4CL1* are located upstream of the phenylpropanoid pathway and are responsible for the removal of an amino group of phenylalanine and the addition of coenzyme A, respectively (Boerjan et al., 2003; Vanholme et al., 2019). The *EjCAD3* gene encodes an enzyme that catalyzes the conversion of coniferyl aldehyde to coniferyl alcohol (Xu et al., 2019). Accordingly, the strong activation of these genes by *EjAGL15* would facilitate the flux of metabolites directed toward monolignol synthesis, which accords with the augmented G and S lignin units observed during loquat fruits’ postharvest storage. Despite its role in lignin biosynthesis, *EjAGL15* also regulates lignin monomer polymerization to some extent. Interestingly, *EjAGL15* impairs the promoter activity of *EjPRX12*, which is inconsistent with other monolignol biosynthesis-related genes. Given the molecular functions of enzyme-encoding genes that *EjAGL15* activates, the expression of *EjAGL15* should lead to the accumulation of coniferyl alcohol and sinapyl alcohol, both being substrates for lignin polymerization. Nevertheless, the repression of *EjPRX12*’s promoter indicates a negative effect on the polymerization of monolignols, which seems contrary to the lignin content increasing during the postharvest storage period. This contradiction may be explained by the redundancy of genes that encode peroxidase. It has been confirmed that multiple PRXs are associated with lignin content in the stem tissue of Arabidopsis, such as *AtPRX52* (Fernandez-Perez et al., 2015b), *AtPRX71* (Shigeto and Tsutsumi, 2016), and *AtPRX72* (Herrero et al., 2013; Fernandez-Perez et al., 2015a). In loquat, EjPRX12 is able to specifically catalyze sinapyl alcohol (Zhang et al., 2022), and other lignin-related PRX genes have yet to be reported. Considering our findings that the G lignin content continually increases during loquat storage, but EjPRX12 does not use coniferyl alcohol as a substrate, we may infer that other genes encoding peroxidase are required for biosynthesis of the G unit. This circumstantial evidence proves that *EjPRX12* alone is insufficient for complete polymerization of all lignin units present in loquat flesh. Meanwhile, those genes responsible for lignin biosynthesis are strongly activated by *EjAGL15* and this leads to the accumulation of monolignols. Hence, the sole inhibition of *EjPRX12* may not slow the rate of monolignol polymerization enough to significantly reduce the flesh lignin content.

Apart from genes in the phenylpropanoid pathway, *EjAGL15* also affects multiple regulators of loquat flesh lignification (**Figure 5B**). According to the luciferase assay, the EjAGL15 had an activation or repression effect on the promoters of MYB, AP2, and NAC family members. Both *EjMYB2* and *EjMYB8* are significantly up-regulated by EjAGL15 whereas *EjMYB1* and *EjAP2*-1 are repressed. Interestingly, the regulatory effects of EjAGL15 on different lignification regulators does not lead to a consistent influence upon *Ej4CL1*, which is the direct target of *EjMYB1*, *EjMYB2*, and *EjMYB8*. For instance, *EjMYB2* and *EjMYB8* are a repressor and activator of *Ej4CL1*, respectively, and yet both are activated by *EjAGL15.* In general, *EjAGL15* functions more like a positive regulator of flesh lignification due to its strong activation of genes encoding lignin biosynthesis-related enzymes and its significant positive correlation with lignin content.

Although *EjAGL15* has biological functions vis-à-vis multiple lignin-related genes, the direct linkage between *EjAGL15* and them or regulators of lignification have not been established. Intriguingly, the abundance of *EjMYB8* and *EjERF39* transcripts remained at a markedly low level at 20°C versus 0°C (Zhang et al., 2020), which presumably limits their activation effect on *Ej4CL1*’s promoter. Hence, we suspect the strong activation on *Ej4CL1* by *EjAGL15* should be mediated by genes other than *EjMYB8* and *EjERF39*, this corresponding to the existence of a senescence-specific signal transduction routine in which *EjAGL15* acts as one of the key nodes.

### 4.2 *EjAGL15* is involved in senescence-induced flesh lignification

In loquat fruits, lignin gets deposited in their flesh during postharvest storage at either 20°C or 0°C and the responsible mechanisms are thought differ (Yang et al., 2010). Generally, senescence and chilling injury are considered crucial factors that account for the postharvest lignification of loquat at 20°C and 0°C, respectively, in that both processes entail genes being activated in the phenylpropanoid pathway, such as *Ej4CL1* and *EjCAD5* (Li et al., 2017; Xu et al., 2019). According to previous reports, the lignin deposition in loquat flesh in response to chilling injury is mainly regulated by various transcription factors, such as those in the MYB family (Xu et al., 2014; Zhang et al., 2020); however, it remains unclear whether senescence-induced lignin deposition is likewise under control of that same set of genes. Actually, transcript profiles during loquat fruit’s storage at 20°C have not been characterized for most of its transcription factors active in the regulation of chilling-induced flesh lignification, with the exception of *EjMYB8* and *EjERF39*. However, the abundance of *EjERF39* and *EjMYB8* transcripts is low during storage at 20°C (Zhang et al., 2020), indicating their absence in modulating lignin deposition during senescence. The limited results discovered previously left it difficult to reliably identify key regulators of senescence-induced lignin deposition in loquat flesh. In this study, we discovered a MADS-box gene, *EjAGL15*, whose transcription was induced during storage at 20°C but at 0°C it declined continually. The expression pattern of *EjAGL15* indicates this gene has a close relationship with senescence, as confirmed by the Pearson correlations with the flesh lignin data. Further, *EjAGL15* probably fulfills its function only at 20°C given the low abundance of its gene transcripts at 0°C. Collectively, these clues indicate that *EjAGL15* drives a rapid augmentation of lignin content under loquat fruit's storage at 20°C, but not so at 0°C. Thus, *EjAGL15* tends to activate lignin biosynthesis via senescence-induced lignification rather than a reliance on chilling.

The senescence-related expression pattern of *EjAGL15* is consistent with its homologs in Arabidopsis. The Arabidopsis *AGL15* is a repressor of senescence and overexpressing this gene leads to the increase of perianth longevity (Fang and Fernandez, 2002). The delay in perianth abscission originates from the repression of abscission-related genes, like the receptor-like protein kinases *HAESA*, by AGL15 in Arabidopsis (Patharkar and Walker, 2015). On the other hand, *AGL15* has been suggested to function as a regulator in lignified tissue formation (Cosio et al., 2017). Although it could be hypothesized that *AGL15* postpones the senescence-induced abscission of perianth by regulating lignin deposition in the abscission zone, the detailed mechanism by which *AGL15* manipulates lignin deposition has not been fully revealed. Thus, it is hard to predict the exact targets of *EjAGL15* based on its Arabidopsis homologs. Here, we identified several potential targets of the *EjAGL15*, including *Ej4CL1*, *EjCAD3*, *EjCAD4* and *EjPRX12*, which explains how senescence can trigger flesh lignification in loquat. *EjPRX12* most resembles the homolog of Arabidopsis *PRX17*, which is reportedly under regulation by *AGL15*. Similarly, in this study, we find that *EjAGL15* significantly inhibits the promoter of *EjPRX12*. However, the activation effect of *EjAGL15* upon lignin biosynthesis-related genes such as *Ej4CL1*, *EjCAD3*, and *EjCAD4* has not been found in Arabidopsis. Our data indicate *EjAGL15* may regulate not only polymerization but also the biosynthesis of monolignol. The discovery of *EjAGL15* provides a node that had been missing in the complex network used to regulate senescence-induced lignification, making it now possible to screen other genes that work synergistically or antagonistically with *EjAGL15*.

### 4.3 The discovery of *EjAGL15* extends our understandings of the regulatory mechanism of lignin-related MADS-box gene in loquat

Generally, MADS-box genes are considered to operate as regulators of flower morphogenesis or pollen maturation (Michaels and Amasino, 1999; Yu et al., 2014). For example, the loquat MADS-box genes *EjCAL* can promote the flower bud differentiation process (Xu et al., 2022). The connection between MADS-box genes and fruit lignification could not be established until the loquat *EjAGL65* was first identified (Ge et al., 2021). The *EjAGL65* gene is responsive to chilling injury but not senescence, and it negatively regulates lignin biosynthesis. As the second lignin-related MADS-box gene found in loquat, *EjAGL15* differs from *EjAGL65* in many aspects. Firstly, *EjAGL15* is not a member of the Mδ subgroup to which EjAGL65 belongs. Through phylogenetic analysis we find that *EjAGL15* is assigned to the MIKC subgroup of Arabidopsis, as per the classification by Parenicova et al. (2003). Members of that MIKC subgroup have been proven to play pivotal roles in plant development (Gramzow and Theissen, 2010; Schilling et al., 2020) and plant responses to stress (Arora et al., 2007; Jia et al., 2018). Interestingly, *EjAGL15* is a close homolog of Arabidopsis *AGL15*, which participates in age-dependent lignified tissue formation (Cosio et al., 2017). Overexpressing *AGL15* leads to the deposition of lignin in the dehiscent zone of petal and sepal parts in Arabidopsis, thus delaying the senescence of perianth (Fang and Fernandez, 2002). The results of the present study imply a similar role for *EjAGL15* as a positive regulator of lignin deposition. But the increased abundance of *EjAGL15* transcripts in flesh tissue results in the deposition of lignin in the same area, which unexpectedly changed the function of *EjAGL15*: from delaying senescence to deteriorating fruit quality. Secondly, *EjAGL15* was able to activate lignin biosynthesis-related genes and its transcript abundance is positively correlated with lignin content, which suggests an effect opposite that exerted from *EjAGL65* on regulating flesh lignification. In addition, *EjAGL15* exacts a biological effect on multiple targets in the phenylpropanoid pathway, but the target of *EjAGL65* is limited to *Ej4CL1*. Furthermore, *EjAGL15* and *EjAGL65* are induced by different storage temperatures and regulate lignin deposition under differing physiological disorders. All the above lines of evidence point to *EjAGL15* and *EjAGL65* respectively having a positive and negative influence upon flesh lignin deposition, for which the genes recruited by them for transducing the signal from senescence and chilling injury also differ. Considering the fact that the lignin content of loquat fruit flesh increased so rapidly under the 20°C treatment condition, in tandem with key regulators of chilling-induced lignification like *EjMYB8* and *EjERF39* not being highly transcribed, *EjAGL15* probably activates lignin biosynthesis via a pathway that is independent of the currently discovered transcription factors. Altogether then, the MADS-box family genes *EjAGL15* and *EjAGL65* are deeply involved in manipulating senescence- and chilling-induced lignin deposition, respectively.

## 5 Conclusion

This study identified a MADS-box gene, *EjAGL15*, induced by senescence. *EjAGL15* belongs to the MIKC subgroup of the MADS-box family and shows a positive correlation with the lignin content of loquat fruit flesh. The dual-luciferase assay revealed *EjPAL1*, *Ej4CL1*, *EjCAD3*, *EjCAD4*, and *EjPRX12* as potential targets of *EjAGL15*. Similar to its Arabidopsis homolog *AGL15*, *EjAGL15* functions in facilitating lignin deposition. However, in contrast to *AGL15* which delays the abscission of perianth by depositing lignin in the dehiscent zone, *EjAGL15* expression is induced in the flesh of loquat fruit, contributing to the deterioration of its fruit quality during storage.

## Data availability statement

The datasets presented in this study can be found in online repositories. The names of the repository/repositories and accession number(s) can be found below: https://www.ncbi.nlm.nih.gov/genbank/, OQ435240.

## Author contributions

JC and HG conceived the study and supervised the experiments. HG and XL performed the experiments and analysis. HG wrote the article. HX and JC was involved in the discussion of the manuscript organization and revised the manuscript. All authors contributed to the article and approved the submitted version.
