# Supplementary material for: The MADS-box gene EjAGL15 positively regulates lignin deposition in the flesh of loquat fruit during its storage
Source: Front Plant Sci. 2023 May 10;14:1166262. doi: 10.3389/fpls.2023.1166262 (PMC10205988; doi:10.3389/fpls.2023.1166262)
Supplement: Supplementary file 1 [file DataSheet_1.docx]

Supplementary Material

The MADS-box gene *EjAGL15* positively regulates lignin deposition in the flesh of loquat fruit during its storage

**Hang Ge, Hongxia Xu, Xiaoying Li, Junwei Chen^*^**

*** Correspondence:**

Junwei Chen

Email: chenjunwei@zaas.ac.cn

# Supplementary Figures and Tables

## Supplementary Figures


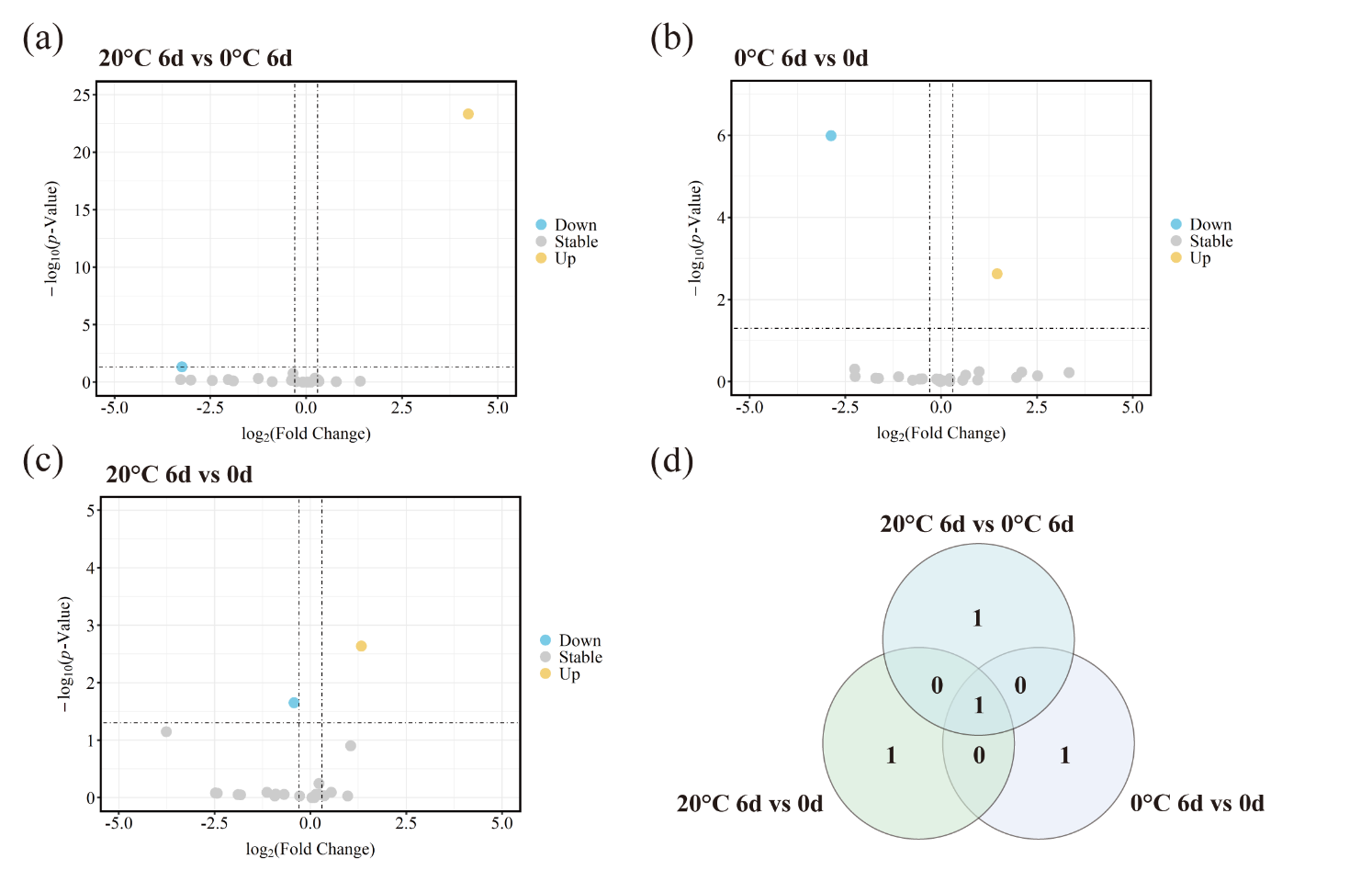


**Supplementary Figure 1** Characterization of the differentially expressed MADS-box genes by transcriptome analysis. (a–c) Differentially expressed MADS-box genes were visualized using volcano plots drawn between flesh samples stored for 6 days under 20°C and 0°C (a), those stored for 6 days under 0°C and before storage (b), those stored for 6 days under 20°C and before storage (c). (d) Venn plot of differentially expressed MADS-box genes that had been mined from the transcriptome data.

**
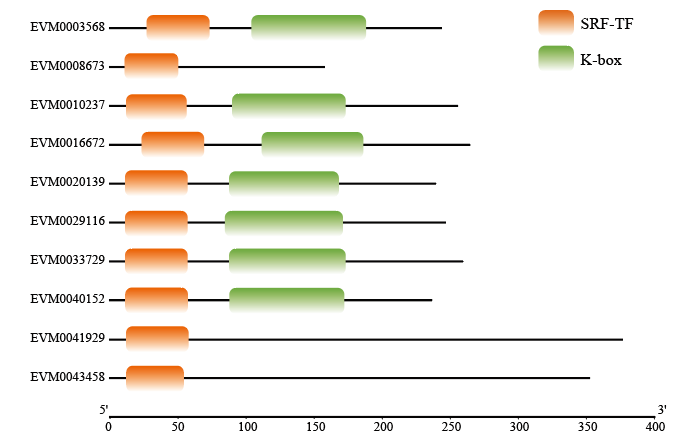
**

**Supplementary Figure 2** Distribution of Conserved domains detected in the amino acids sequences of 10 MADS-box genes. The orange color represents the SRF domain which locates at N terminal and is the symbol of MADS-box members. Green box represents K-box domain which is conserved in MIKC subgroup of MADS-box genes.


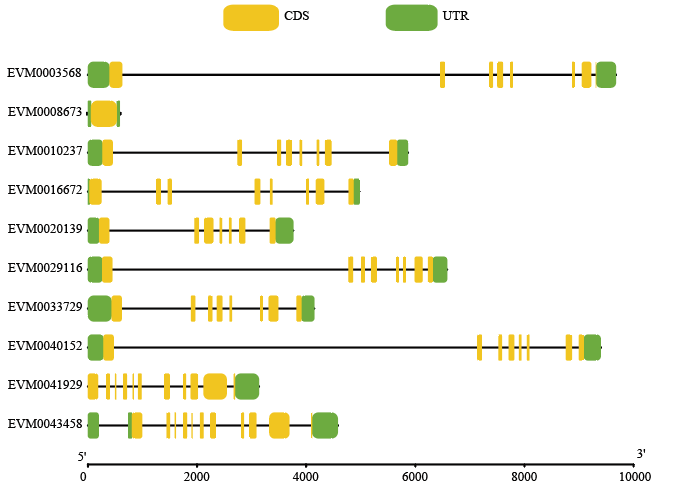


**Supplementary Figure 3** Gene structure of 10 MADS-box genes. Untranslated regions (UTR) were labelled in green, and exons were in yellow. Lines between colored boxes indicate the introns.

## Supplementary Tables

**Table S1** Primers used for quantitative reverse transcription PCR

| **Gene ID** | **5’ Primer** | **3’ Primer** |
| --- | --- | --- |
| EVM0033729 | CCATGGCGTCACAAGTAGTG | ATTGGCTCCCTGAGTCATTG |
| EVM0043458 | GTGGGCACATTCCCTATCTT | TCTAGGAGCTTCAAAGCCATT |
| EVM0029116 | GGTACTCTGCAGTGGGGTCT | AGCATAAACTTGGGCATGCT |
| EVM0020139 | AGCAGAACATTGGCCTTGAC | TCTTTCCATTTAGTTCTTCCACC |
| EVM0003568 | CAGCTCCTACGAGCAAAGATAG | TGAGACTGCAGGGTCTCATA |
| EVM0040152 | GTCAACGAAGGCCCAATTTATG | AGCCTGGTGATTGCTATTCC |
| EVM0008673 | CTGACTGGTTGCAGAATTTGAG | CACGCCTCTTGCATTTCTTC |
| EVM0010237 | GGTCCTATTACCAGGCCATTAG | GCAGCAGCATCTTAGGTCTAT |
| EVM0016672 | TCCAGTTACGAAGCAGAAGAAA | CTTGACTCCCTGAATCGTTAGAA |
| EVM0041929 | AGGAAGCGGTGGCTTAAAT | CTGGCACGTAAGAGCTACAATA |

**Table S2** Primers used for amplifying full coding sequence of *EjAGL15*

| **Gene ID** | **5’ Primer** | **3’ Primer** |
| --- | --- | --- |
| EVM0033729  (*EjAGL15*) | ATGGGAAGGGGGAAGATTGA | TTACAGACCTAATTGGCTCC |

**Table S3** Bayesian Information Criterion (BIC) score of different models that describing the substitution pattern, the model with highest BIC score was used for tree construction.

| **Model** | **#Param** | **BIC** | **AICc** |
| --- | --- | --- | --- |
| JTT+G+F | 271 | 112544.2468 | 110277.8312 |
| JTT+G+I+F | 272 | 112554.627 | 110279.8654 |
| JTT+G | 252 | 112785.1024 | 110677.2847 |
| JTT+G+I | 253 | 112795.4826 | 110679.3165 |
| LG+G+F | 271 | 113350.9087 | 111084.4932 |
| WAG+G+F | 271 | 113361.244 | 111094.8284 |
| LG+G+I+F | 272 | 113361.2889 | 111086.5274 |
| WAG+G+I+F | 272 | 113371.6241 | 111096.8626 |
| LG+G | 252 | 113532.0529 | 111424.2351 |
| LG+G+I | 253 | 113542.4331 | 111426.2669 |
| WAG+G | 252 | 113749.4642 | 111641.6465 |
| WAG+G+I | 253 | 113759.8444 | 111643.6783 |
| Dayhoff+G+F | 271 | 113770.2718 | 111503.8562 |
| Dayhoff+G+I+F | 272 | 113780.652 | 111505.8904 |
| rtREV+G+F | 271 | 113800.1023 | 111533.6867 |
| rtREV+G+I+F | 272 | 113810.4824 | 111535.7209 |
| Dayhoff+G | 252 | 114483.2611 | 112375.4433 |
| Dayhoff+G+I | 253 | 114493.6413 | 112377.4751 |
| rtREV+G | 252 | 114543.9975 | 112436.1798 |
| rtREV+G+I | 253 | 114554.3777 | 112438.2116 |
| mtREV24+G+F | 271 | 114571.9679 | 112305.5523 |
| mtREV24+G+I+F | 272 | 114582.3481 | 112307.5865 |
| cpREV+G+F | 271 | 115137.8244 | 112871.4088 |
| cpREV+G+I+F | 272 | 115148.2046 | 112873.443 |
| cpREV+G | 252 | 115304.7961 | 113196.9783 |
| cpREV+G+I | 253 | 115315.1763 | 113199.0101 |
| JTT+F | 270 | 115343.7615 | 113085.692 |
| JTT+I+F | 271 | 115354.1418 | 113087.7262 |
| JTT | 251 | 115458.2033 | 113358.7341 |
| JTT+I | 252 | 115468.5836 | 113360.7659 |
| WAG | 251 | 115915.2742 | 113815.805 |
| WAG+I | 252 | 115925.6544 | 113817.8366 |
| WAG+F | 270 | 116103.2302 | 113845.1608 |
| WAG+I+F | 271 | 116113.6104 | 113847.1948 |
| LG | 251 | 116420.1034 | 114320.6342 |
| LG+F | 270 | 116427.0478 | 114168.9783 |
| LG+I | 252 | 116430.4836 | 114322.6658 |
| LG+I+F | 271 | 116437.428 | 114171.0124 |
| Dayhoff+F | 270 | 116535.8867 | 114277.8172 |
| Dayhoff+I+F | 271 | 116546.2667 | 114279.8511 |
| rtREV+F | 270 | 116687.3661 | 114429.2966 |
| rtREV+I+F | 271 | 116697.7463 | 114431.3307 |
| Dayhoff | 251 | 117024.7962 | 114925.327 |
| Dayhoff+I | 252 | 117035.1764 | 114927.3587 |
| rtREV | 251 | 117197.7988 | 115098.3296 |
| rtREV+I | 252 | 117208.179 | 115100.3613 |
| mtREV24+G | 252 | 117586.709 | 115478.8912 |
| mtREV24+G+I | 253 | 117597.0892 | 115480.923 |
| mtREV24+F | 270 | 117731.5405 | 115473.471 |
| mtREV24+I+F | 271 | 117741.9206 | 115475.5051 |
| cpREV+I | 252 | 118246.8682 | 116139.0505 |
| cpREV | 251 | 118313.9178 | 116214.4486 |
| cpREV+F | 270 | 118399.0939 | 116141.0244 |
| cpREV+I+F | 271 | 118402.2437 | 116135.8282 |
| mtREV24 | 251 | 120916.553 | 118817.0838 |
| mtREV24+I | 252 | 120926.9332 | 118819.1155 |
